# Testing the ‘Extreme Female Brain’ Theory of Psychosis in Adults with Autism Spectrum Disorder with or without Co-Morbid Psychosis

**DOI:** 10.1371/journal.pone.0128102

**Published:** 2015-06-12

**Authors:** Felicity V. Larson, Meng-Chuan Lai, Adam P. Wagner, MRC AIMS Consortium, Simon Baron-Cohen, Anthony J. Holland

**Affiliations:** 1 Cambridge Intellectual and Development Disabilities Research Group, Department of Psychiatry, University of Cambridge, Cambridge, United Kingdom; 2 Autism Research Centre, Department of Psychiatry, University of Cambridge, Cambridge, United Kingdom; 3 Department of Psychiatry, National Taiwan University Hospital and College of Medicine, Taipei, Taiwan; 4 NIHR CLAHRC East of England, Cambridge, United Kingdom; 5 Cambridgeshire and Peterborough NHS Foundation Trust, Cambridge, United Kingdom; Nagoya University Graduate School of Medicine, JAPAN

## Abstract

**Introduction:**

Males and females in the general population differ, on average, in their drive for empathizing (higher in females) and systemizing (higher in males). People with autism spectrum disorder (ASD) show a drive for systemizing over empathizing, irrespective of sex, which led to the conceptualisation of ASD as an ‘extreme of the typical male brain’. The opposite cognitive profile, an ‘extreme of the typical female brain’, has been proposed to be linked to conditions such as psychosis and mania/hypomania.

**Methods:**

We compared an empathizing-over-systemizing bias (for short ‘empathizing bias’) in individuals with ASD, who had experienced psychotic illness (N = 64) and who had not (N = 71).

**Results:**

There were overall differences in the distribution of cognitive style. Adults with ASD who had experienced psychosis were more likely to show an empathizing bias than adults with ASD who had no history of psychosis. This was modulated by IQ, and the group-difference was driven mainly by individuals with above-average IQ. In women with ASD and psychosis, the link between mania/hypomania and an empathizing bias was greater than in men with ASD.

**Conclusions:**

The bias for empathizing over systemizing may be linked to the presence of psychosis in people with ASD. Further research is needed in a variety of clinical populations, to understand the role an empathizing bias may play in the development and manifestation of mental illness.

## Introduction

Baron-Cohen [1] defined two mental domains: empathizing and systemizing. According to Baron-Cohen, empathy is “our most powerful way of understanding and predicting the social world” ([1] p. 248). In contrast, systemizing is defined as an inductive process governed by laws, patterns, and logic. It is integral for the understanding of systems and non-human elements of the universe [1].

Baron-Cohen [1] was the first to link differences at a group level between males and females on the dimensions of empathizing and systemizing to autism spectrum disorder (ASD). This ‘extreme male brain’ (EMB) theory argues that the cognitive characteristics of ASD are an extreme of the typical male cognitive style–they have an ‘extreme’ drive for systemizing over empathizing. The EMB hypothesis drew on a broad range of experimental findings, as well as observations by Asperger [2], of a link between what he called ‘male intelligence’ and ASD. The argument for an association between EMB and ASD has been strengthened by findings that people with ASD show a stronger drive for systemizing, as measured by the Systemizing Quotient (SQ) [3], and a reduced drive for empathizing, measured by the Empathy Quotient (EQ) [4], relative to controls. These results have been recently replicated in large samples [5]. The biological underpinnings of the EMB hypothesis are not yet clear but are being actively investigated [6–9].

Baron-Cohen [1] also describes the ‘extreme female brain’ (EFB), a proposed opposite profile to the EMB. The EFB is described as a cognitive style that is challenged in understanding systems, for example due to having low mathematical or scientific ability and interest, whilst having an above average drive to empathize. Baron-Cohen [1] proposes that such individuals would function well in societies that value the importance of social interaction and that they may not appear disabled, unlike those with the EMB.

A number of authors have previously linked mentalizing deficits in women to borderline personality disorder [10] and depression [11], presenting early hypotheses regarding the psychiatric presentation of the EFB. However, Crespi and Badcock [12] were the first to depict ASD and psychotic-spectrum disorders as diametrical opposites utilizing the continuum of mentalizing, as well as a range of other evidence including genetics. We will term this “the diametrical ASD-P model”. The spectrum itself is based on the concept of a “social brain” [13–14], which, Crespi and Badcock [12] argue, is hyperactive in psychosis and hypoactive in ASD. This roughly maps onto the ideas of systemizing and empathizing as detailed above.

In line with the diametrical ASD-P model, Brosnan et al. [15] tested if psychotic illness more broadly might represent the EFB, in the same way ASD represents the EMB. This would provide directly measured behavioral-cognitive evidence supporting the model. Crespi and Badcock [12] proposed that if the EMB leads to autism, because of ‘mentalizing’ deficits, the EFB may lead to psychotic illness and paranoia, because of excess and inaccurate mentalizing. Negative symptoms and mentalizing ability are negatively correlated, while positive symptoms of psychotic illness are positively associated with mentalizing ability [16]. The preliminary results reported by Brosnan et al. [15] supported the prediction of an association between certain psychotic traits and a drive for empathizing. However, it was manic features, beyond those generally associated with psychosis or related specifically to schizophrenia, which showed the most significant association in a sample of neurotypical women.

A potential challenge to a diametric model of ASD and psychotic illness would be individuals who have *both* conditions. The presence of people with both conditions is inexplicable within the original diametrical ASD-P model and is one of its key weaknesses. Crespi et al. [17] addressed the complicated genetic relationship between these conditions by proposing multiple ways in which the relationship could be understood. These explanations could equally be extended to the behavioural qualities of ASD and psychosis. Crespi et al. [17] suggest that in some individuals there may be a dichotomous relationship between ASD and schizophrenia, driven by mirrored genetic abnormalities, whilst in other individuals there may be a more complex and subtle relationship.

This challenge to a “pure” dichotomous relationship between ASD and psychosis is not dealt with at a theoretical level by Crespi et al. [17]. Instead, the authors return to the position of the diametrical ASD-P model that any case of apparent co-morbid psychosis and ASD is likely to be a misdiagnosis, rather than a true co-occurrence of two distinct conditions. As an explanation of genetic complexity, this argument perhaps holds appeal. However, there are multiple reports from reputable research groups and clinicians showing that cases of co-morbidity do occur, if possibly rarely (see [18] and [19], for a review]. In the context of mixed genetic and clinical evidence, a more parsimonious explanation is that in at least some cases, ASD and psychosis share causal factors and are thus related in a fundamental biological sense. In turn, this suggested there may be shared behavioral features between the conditions, rather than placing them as opposites on a spectrum.

A second difficulty with the diametrical ASD-P model and its application to the evidence from Brosnan et al. [15] is that some psychiatric conditions (e.g. mania, positive symptoms of psychosis) are *temporary* alterations to a person’s mental state, whereas autistic traits are present throughout life. A more plausible anchor point with obvious similarity to ASD might be the life-long correlate of the temporary state of psychosis: schizotypy, and at the extreme, schizotypal personality disorder (SPD). Similarly to autistic traits, schizotypy can be measured continuously. Schizotypal traits share a well-supported relationship with non-affective psychoses (e.g., [20]), and a partially supported relationship with bipolar disorder [21]. People with SPD show mentalizing deficits similar to those seen in people with schizophrenia [22]. It is unclear why, therefore, the diametrical ASD-P model compares a stable, lifelong condition that develops early in life (ASD) with what could be considered an extreme and acute manifestation of schizotypy (psychosis).

The diametrical ASD-P model has been tested using schizotypal traits, and the evidence is equivocal. One study showed strong support for a continuum between positive schizotypal traits and ASD traits [23] while another, using different measures, showed no support at all [24]. Both studies utilized large but opportunistic undergraduate samples, which again highlights that no direct measures of systemizing or empathizing drive in people with psychosis have been published to date. Both studies used reliable and valid measures of schizotypy and autistic traits, so the reason for the differences remains unclear beyond that the measures may be measuring slightly different constructs.

While the diametrical ASD-P model has undergone some revision, and has been criticised as overly simplistic [25], the idea of a spectrum of individual differences linked to sex can be empirically tested. Is it the case that ASD and psychotic illness (or mania) are extreme examples of male and female cognitive processing biases? With the empirical support for the diametrical ASD-P model being relatively weak, or at the least contradictory, this is clearly an area where more research is needed.

In order to address some of the issues with the EFB highlighted above, we investigated empathizing and systemizing in individuals with a dual diagnosis of ASD and psychotic illness, some of whom also had experienced symptoms of mania or hypomania. In line with Brosnan et al. [15], we chose to focus on psychotic illness more generally rather than schizophrenia specifically. We tested the hypothesis that those with ASD and co-morbid psychosis, particularly those with manic or hypomanic symptoms, would have a higher drive for empathizing over systemizing, compared to individuals with ASD but without psychotic illness. If proved, it would provide support for the hypothesis of a continuum between the EMB and the EFB, expressed by ASD and psychotic illness as diametric opposites. If the hypothesis were not supported, it would cast doubt on Crespi and Badcock’s [12] diametric model. It would also call into question the idea that the sub-clinical manifestations of psychosis or mania used by Brosnan et al. [15] lie on a continuum with what is seen in individuals who have experienced clinically relevant symptoms.

## Method

Ethical approval for the study was given by the Cambridgeshire 3 Research Ethics Committee. All participants were 16 years of age or older (the legal age of consent to participate in research in the United Kingdom) at the time of their involvement and gave informed written consent before participating. Individuals with a clinically confirmed or suspected ASD (DSM-IV/ICD-10 autistic disorder/childhood autism, Asperger’s disorder/syndrome, and pervasive developmental disorder not otherwise specified were all considered as part of the ASD continuum) and a history of psychotic illness of any type were recruited from clinical services across the UK.

All cases of clinically suspected ASD were confirmed by testing using either the Autism Diagnostic Observation Schedule (ADOS) [26], Module 4, or Autism Diagnostic Interview–Revised (ADI-R) [27], with individuals only included if they met all threshold requirements on all scales. Psychotic illness was confirmed using the Diagnostic Interview for Psychosis (DIP) [28], which generates diagnoses using the Operational Criteria Checklist (OPCRIT) algorithms [29], or the Psychiatric Assessment Schedule for Adults with Developmental Disabilities (Mini PAS-ADD) [30]. Features of disorder from the Mini PAS-ADD can also be inputted into the OPCRIT algorithms to generate standardized diagnoses. For all participants, a diagnosis of a disorder mapping to Diagnostic and Statistical Manual of Mental Disorders-IV-TR (DSM-IV-TR) [31] codes 295.xx, 296.x4, 296.4, 296.89, 297.xx, 298.8, and/or 298.9 was present, as defined by at least one of three main diagnostic systems- DSM-IV-TR [31], International Classification of Diseases-10 (ICD-10) [32], and/or the Research Diagnostic Criteria (RDC) [33].

Multiple diagnostic systems were used due to the potential for disagreement between them and the possibility that psychotic illness in people with ASD may present differently, making diagnosis using traditional algorithms difficult. This allowed for a more inclusive approach that did not exclude individuals with clinically relevant symptoms on the basis of any one diagnostic system. All participants had received clinical treatment for psychotic illness at some point and the majority (>90%) were referred by clinical services. The form of the psychosis varied considerably across participants and was not characterised by any particular specific disorder. For example, while some individuals reported paranoid thoughts, this was not a majority. Mood symptoms were common across participants, primarily depression but with additional evidence of mania and/or hypomania. Further details of the full mental health profile of this cohort are being prepared for publication separately [34].

While some participants in the study did report drug and alcohol use around the time of their first onset of psychosis, none were found to meet criteria for substance induced psychosis. At the time of interview, all participants who were directly interviewed were free from overt symptoms of psychosis and were considered by their clinicians or referring individuals to be mentally healthy. In the case of individuals unable to be interviewed directly, status of current symptoms was not important to the study and would not impact on the results presented here. No specific measure of current symptom level was used for this reason.

In total, 65 participants (53 males, 82%) with co-morbid ASD and psychotic illness (the ASD-P group) completed the short forms of the EQ and the SQ (EQ-S, SQ-S). The EQ-S and SQ-S have been validated as reliable short forms of the full EQ and SQ [35], and comprise a subset of the items in the full EQ and SQ and SQ-Revised (SQ-R) [36] questionnaires. Full EQ and SQ-R data from a group of individuals with both clinically and ADI-R, ADOS, or Adult Asperger Assessment [37] confirmed ASD and no known history of psychotic illness were available for comparison (N = 71) (33 males, 47%). They were recruited via the MRC Autism Imaging Multicentre Study (MRC AIMS) project [38–40], and are termed the ASD-No Psychosis (ASD-NP) group. Full-scale IQ scores were available from both groups of participants, collected using the Weschler Abbreviated Scales of Intelligence (WASI) [41]. Additionally, data were available from the OPCRIT [29] for the ASD-P group, allowing for the analysis of manic symptoms and their association with the drive for empathizing over systemizing.

Empathizing bias (EB) is a measure of an overall bias for empathizing over systemizing. It is based on the difference between standardized scores on the EQ and SQ-R (or their short form equivalents). As the scales contain a different number of items, the total scores on each measure are converted to z-scores. The difference between z-scores on these two measures (zEQ − zSQ-R) is the EB score, with a higher score indicating greater drive for empathizing over systemizing [15]. In the current study, to compare the two groups (ASD-P and ASD-NP), the conversions to z-scores were based on a large general population sample (N = 1,761) for which population norms are known for both the full and short forms of the EQ and SQ scales [35–36]. There are very high correlations between the full and short forms of the scales (*r* = .93 for the EQ and EQ-S, and *r* = .95 for the SQ and SQ-S) [35]. Additionally, differences between empathizing and systemizing were used to categorize participants as either extreme systemizers (Extreme Type S), systemizers (Type S), balanced (Type B), empathizers (Type E), or extreme empathizers (Extreme Type E) using the categorizations reported by Wakabayashi et al.[35].

We compared the ASD-P and ASD-NP groups for raw differences on sex split, FSIQ and EB scores (including the categorized form of EB). Regression models were then used to investigate differences in EB scores between the two groups while controlling for the effects of sex and FSIQ. The ASD-P group was examined in greater detail by comparing those with and without mania, first comparing raw differences and then investigating differences in EB having controlled for the effects of sex and FSIQ.

## Results

**Fig 1:**
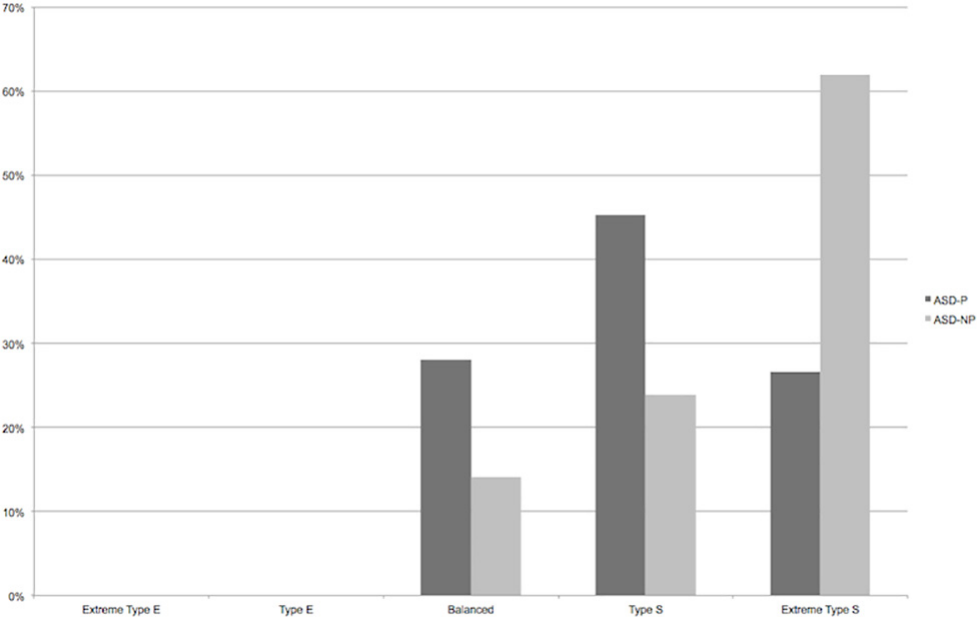
Differences in the distribution of categorical drives for empathizing/systemizing, by group (ASD-P vs ASD-NP).

Raw differences between the groups are reported in Table 1. The ASD-P group had significantly fewer females (ASD-P: 19%; ASD-NP: 54%; *p*<0.001), a significantly lower average FSIQ score (difference = 14, 95% confidence interval (CI): -21, -8, *p*<0.001), and a significantly higher EB score (difference = 1.10, 95% CI: 0.60, 1.59; *p*<0.001). This led to significant differences in the distribution of empathizing-systemizing categories (*p*<0.001; see Fig 1 and Table 1 below), which are derived from the EB scores.

**Table 1:** Raw differences between ASD-P and ASD-NP groups.

	Overall		ASD-psychosis (ASD-P)		ASD-no psychosis (ASD-NP)		Group differences
**N**	135		64		71		–
**Female %**	37		19		54		X²(1) = 15.99, *p*<0.001
**FSIQ mean (SD)**	106	(20)	98	(20)†	112	(16)	Dif = -14 (95% CI = -21, -8), *p*<0.001
**EB mean (SD)**	-2.17	(1.58)	-1.59	(1.06)	-2.69	(1.78)	Dif = 1.10 (95% CI = 0.60, 1.59), *p*<0.001
**Empathizing-systemizing distribution (%)**							
Extreme Type S	45		27		62		
Type S	34		45		24		Fisher's exact test, *p*<0.001
Balanced	21		28		14		
Type E	0		0		0		
Extreme Type E	0		0		0		

† N = 59, due to missing data.

ASD = autism spectrum disorder. FSIQ = full-scale IQ. EB = empathizing bias. SD = standard deviation. Dif = difference. CI = confidence interval.

The initial regression model relating EB to sex, FSIQ, group membership (ASD-P vs ASD-NP) and their second order interactions showed that sex × FSIQ and sex × group interactions were not significant (b = -0.02, *p* = 0.208 and b = 0.72, *p* = 0.199 respectively; model not reported). A new regression model was fitted which excluded these non-significant interactions, resulting in the model reported in Table 2 (R² = 0.33).

**Table 2:** Regression model fit to empathizing bias (EB), across both groups (N = 130, given the missing values on FSIQ–see Table 1).

Term	b	Standard error b	95% confidence interval		*p*
**Intercept**	-0.40	–	–		–
**Female†**	-0.44	0.26	-0.94,	0.07	0.092
**FSIQ**	-0.01	0.01	-0.03,	0.01	0.204
**ASD-NP‡**	4.41	1.41	1.62,	7.20	0.002
**FSIQ × ASD-NP**	-0.05	0.01	-0.07,	-0.02	<0.001

Interaction is denoted by ‘×’.

† Male taken as reference level.

‡ ASD-P taken as reference level. FSIQ = full scale IQ.

FSIQ had a significantly stronger effect in the ASD-NP group (FSIQ×ASD-NP, *p*<0.001), decreasing EB by 0.06 for each point increase in FSIQ; in the ASD-P group, EB decreased by only 0.01 for each point increase in FSIQ. Sex did not have a significant effect at the 5% level (*p* = 0.092). Imputing the missing FSIQ values (replacing the five missing FSIQ values, which all occur in the ASD-P group, with the mean ASD-P FSIQ score) and repeating the above analysis gave very similar results.

**Fig 2:**
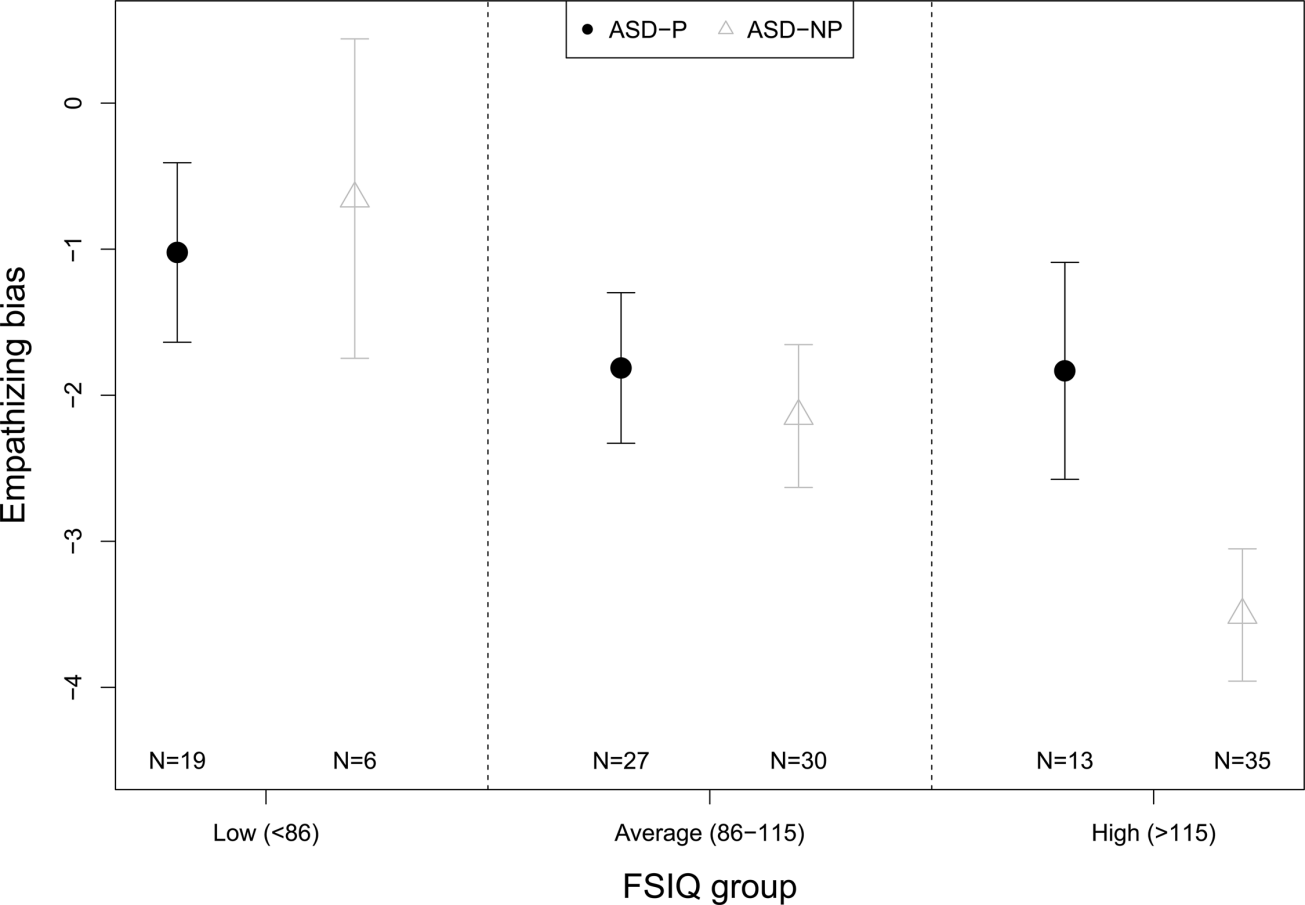
Fitted empathizing bias (EB) means. This figure shows fitted EB means with 95% confidence intervals, within each group at each level of the categorized full-scale IQ.

Given the interaction between group membership and FSIQ, it is difficult to interpret differences in EB between ASD-P and ASD-NP. Thus, to aid interpretation, we categorized FSIQ into three groups based on population norms (mean = 100 and standard deviation (SD) = 15): low (less than 85, more than 1 SD below the mean), average (85 to 115, a two SD range, centred on the mean) and high (>115, over 1 SD above the mean). We then fitted a regression model that included terms for (categorized) FSIQ, group membership and their interaction (a similar model to that reported in Table 2, but excluding sex and using the categorical form of FSIQ). The fitted means, with 95% CI, within each group at each of the categorical levels of FSIQ are shown in Fig 2. There was a clear difference between groups at the high level of FSIQ, with ASD-NP scoring lower than ASD-P. The approach of categorising a continuous variable into groups defined by SD distance from a mean to explore a continuous/categorical interaction has been illustrated by O’Connor [42] and is described in Aiken and West [43], and Cohen and Cohen [44].

Raw differences between those in the ASD-P group with and without cardinal symptoms of mania or hypomania are reported in Table 3. There were no significant differences.

**Table 3:** Raw differences between those with and without mania/hypomania, within the psychotic group.

	Psychosis & no mania/hypomania		Psychosis & mania/hypomania		Group differences
**N**	38		26		-
**Female %**	16		23		Fisher's exact test, *p* = 0.525
**FSIQ mean (SD)**	95	(19)†	102	(21)‡	Dif = -7 (95% CI = -18, 4), *p* = 0.224
**EB mean (SD)**	-1.51	(0.98)	-1.71	(1.17)	Dif = 0.20 (95% CI = -0.37, 0.76), *p* = 0.484
**Empathizing-systemizing distribution (%)**					
Extreme Type S	24		31		
Type S	42		50		Fisher's exact test, *p* = 0.418
Balanced	34		19		
Type E	0		0		
Extreme Type E	0		0		

† N = 36, due to missing data.

‡ N = 23, due to missing data. FSIQ = full-scale IQ. EB = empathizing bias. SD = standard deviation. Dif = difference. CI = confidence interval.

Within the ASD-P group, an initial regression model relating FSIQ, sex and presence of mania or hypomania (yes/no) and their second order interactions to EB showed that sex × FSIQ and FSIQ × mania interactions were not significant (b = 0.005, p = 0.846 and b = -0.002, *p* = 0.901 respectively; model not reported). A new regression model was fitted which excluded these non-significant interactions, resulting in the model reported in Table 4 (R² = 0.21). FSIQ did not have a significant effect (b = -0.01, *p* = 0.448). The presence, or not, of mania had a larger effect on EB in women compared to men (female × mania/hypomania present, b = 1.63, *p* = 0.02). While this is a relatively large effect (using ASD-P EB SD from Table 1, d = 1.63/1.06≈1.5), it should be interpreted with caution as there were only a small number of women in the ASD-P group (12 in ASD-P: six with mania/hypomania and six without).

**Table 4:** Regression model fit to empathizing bias (EB) within the psychotic group (N = 59 given the missing values on FSIQ–see Table 3).

Term	b	Standard error b	95% confidence interval		*p*
**Intercept**	-0.72	–	–		–
**FSIQ**	-0.01	0.26	-0.02,	0.01	0.448
**Female†**	-1.56	0.01	-2.50,	-0.62	0.002
**Mania/hypomania present‡**	-0.45	1.41	-1.08,	0.19	0.164
**Female × Mania/hypomania present**	1.63	0.01	0.25,	3.00	0.021

Interaction is denoted by ‘×’.

† Male taken as reference level.

‡ Presence of no mania taken as reference level. FSIQ = full scale IQ.

## Discussion

It has been proposed that psychotic illness, and in particular mania or hypomania, is linked to an increased drive for empathizing over systemizing [12, 15]. This theory was tested in a group of people with ASD, some of whom had a co-morbid diagnosis of psychotic illness. The evidence from our study suggests some support for an attenuation of the extreme cognitive bias for systemizing over empathizing generally seen in people with ASD when they have co-occurring psychotic illness. This was shown both in the context of EB scores when confounding factors were controlled for, as well as in uncorrected comparisons of numbers of individuals in the Type B, Type S, and Extreme Type S categories. In each case, the ASD-P group showed a greater bias for empathizing over systemizing than the ASD-NP group. The driver of this difference was individuals with above-average IQ scores, who in the ASD-P group had a great bias for empathizing over systemizing than in the ASD-NP group. However, it is important to note that all participants across both groups achieved at most a balanced preference, with a clear bias towards systemizing as a whole, as one would expect from people with ASD irrespective of sex and age [5, 45].

Why there was a significant difference in EB between ASD-P and ASD-NP groups only in those with above-average IQ is interesting. In a sample of neurotypical male adults, Lai and colleagues [38] found negligible correlations between ‘D score’ (equivalent to EB but opposite in sign) and verbal IQ (r = −0.007, *p* = 0.95), performance IQ (r = −0.005, *p* = 0.97) or full-scale IQ (r = 0.002, *p* = 0.99). Published studies to date, however, have not addressed the question of whether IQ has any association with D score (or EB) in individuals with ASD. It does not appear as though IQ has an effect on EB in all individuals with ASD. The presence of psychosis appears to attenuate the positive association between IQ and EB. Previous research into the relationship between psychosis and drive for empathizing [15] did not consider IQ in the interpretation of results. Our study suggests that this may be a further factor to be considered, particularly when studying ASD.

Our finding suggests that the diametrical ASD-P model is too simplistic to fully explain the factors involved, even if there is a relationship between overall drive for empathizing and psychotic illness and/or mania/hypomania. Individuals with a dual-diagnosis of ASD and psychosis should not exist, according to the diametrical ASD-P model. The cohort in this study show clear evidence of pre-morbid ASD traits, measured using gold-standard instruments, and clear evidence of psychotic symptoms, evidenced by fulfilling criteria for psychosis, measured with standardised instruments. In the authors’ experience of these individuals, the psychosis in each case stood separate and distinct from the pre-morbid ASD, and full details of the phenomenology of this cohort are being prepared for publication to further address this point [34].

Our data indicate that a drive for empathizing over systemizing might be more strongly linked to manic or hypomanic symptoms in women with ASD and co-occurring psychotic illness, compared with men with the same conditions. However, the small number of females in the ASD-P group makes it difficult to draw firm conclusions. It should be noted that even those with mania or hypomania in the present study could not be described as Type E or Extreme Type E–at most, a Type B profile resulted, as one would expect for people with ASD [5, 45]. We conclude from this that the systemizing-over-empathizing profile of women with ASD (compared to neurotypical women in the general population) may be reduced in the presence of symptoms of mania/hypomania, which may provide partial support for the findings of Brosnan et al. [15]. The sex-differential effect, however, requires more investigation.

The present results suggest that while factors leading to the development of ASD may set the balance of the brain towards systemizing over empathizing, this balance is also affected by factors involved in the development of psychotic illness in individuals with ASD. That is, individuals with both ASD and psychotic illness have a less strong bias for systemizing over empathizing than is generally seen in people with ASD. However, it is clearly not the presence of an extreme bias towards empathizing over systemizing that has led to vulnerability to psychosis in this group, as the diametrical ASD-P model suggests.

We also speculate that those with ASD and psychotic illness may be different from individuals with psychotic illness but without ASD, when it comes to empathizing-systemizing bias. Differences in proxies for psychotic thinking have been reported in adolescents with ASD [46] compared with findings using a similar measure administered in a large general population cohort [47]. However, to date there are no published accounts of an empathizing over systemizing bias in a cohort of individuals with psychotic illness who do not have ASD. Given that rates of ASD found in populations with psychotic illness may be higher than in the general population [48], any such study would need to account for the effect of underlying autistic traits, which have been linked to a preference for systemizing over empathizing [5, 45].

A more valid adaptation of the diametrical ASD-P model, as discussed in the Introduction, may be to place ASD and SPD at opposite ends of a spectrum. However, recent evidence has failed to equivocally support the presence of an empathizing bias linked to higher schizotypy and lower autistic traits [23–24]. More research would be required, again with individuals at the extreme ends of the continuums (those with ASD and those with SPD), to test this theory further. Additionally, many of the measures used to quantify ASD in relation to empathizing/systemizing drive in previously published studies have been robust but fairly limited in detail, such as the Autism Spectrum Quotient (AQ) [49]. A strength of the current study is the use of a well-validated and extensive measure of ASD (the ADI-R) analysed in relation to empathizing/systemizing drive. Future studies should endeavour to provide more detail about both autistic and schizoptypal traits in order to fully explore what is clearly a complex relationship.

Additionally, it could be the case that psychotic illness and/or SPD do not represent a manifestation of the EFB, and that the diametrical ASD-P model is thus not supported. Little is known about the long-term stability of empathizing/systemizing drives in any population. This raises the possibility that psychotic illness itself changes one’s bias for empathizing or systemizing, rather than the bias for empathizing over systemizing being involved causally in the development of psychotic illness as the diametrical ASD-P model predicts. Longitudinal research into empathizing/systemizing drives over time, therefore, would also be warranted to help explore any causal relationship between extreme drives and psychopathology. Once again, studies in individuals with clinically relevant difficulties, rather than sub-clinical traits in general population samples, would be most useful.

It seems likely that some other factor, beyond schizotypy or diagnosis of psychotic illness, may be linked to a bias for empathizing over systemizing. Even among the individuals presented in this study, there is likely to be a wide range of variability across a number of dimensions related to psychiatric conditions. Mood instability is one such dimension that is theoretically linked to the EFB and may be an alternative to psychosis or schizotypy in a model of understanding the EFB. This is supported in part by the data from Brosnan et al. [15] linking higher empathizing bias to more manic traits. Also, the theoretical links between extreme preference for empathy and borderline personality disorder [10] and depression [11] deserve consideration. Borderline personality disorder, in particular, is characterised by difficulties with affect regulation [32]. However, it would be problematic to place affective instability/dysregulation opposite ASD on any spectrum, given what is known about affect regulation difficulties in people with ASD [50–51] and rates of affective illnesses such as bipolar disorder in people with Asperger’s syndrome in particular [52]. Based on these ideas and the evidence presented in this paper, we propose that the links between affect dysregulation/instability and the EFB should be explored in future research as an alternative to the diametrical ASD-psychosis model.

It is important to acknowledge some limitations of the current study. First, the sample sizes are relatively small for questionnaire-based studies. However, given the confined sample selection (especially for the ASD-P group) the current sample size is reasonable. It may be that there are other differences of smaller effect between the ASD-P and ASD-NP groups that were not detected due to insufficient statistical power resulting from the moderate sample sizes. Another concern may be that for the ASD-P group, the short forms of the EQ and SQ were used, while the ASD-NP group completed the full EQ and SQ-R. We consider this less likely to be an issue statistically, given the established psychometric equivalence of the two measures and their very high levels of agreement [35] and given our use of established general population means in calculating the z-scores [36] for generating the target outcome variable (EB). Lastly, it could be argued that the two groups in this study were not matched rigorously and that the comparison sample represents a sample of convenience. However, it is unclear to us how individuals with ASD with and without psychotic illness could be matched, given the huge range of variability across a number of dimensions that is found amongst people with ASD [53–54], despite the shared core domain deficits necessary for a diagnosis of ASD. Instead, we chose to control for differences between the groups statistically. A key strength of this study, on the other hand, is its use of a unique population of individuals with co-occurring ASD and psychotic illness, who are under-represented in previous studies.

Our results offer some support for the idea that there is a link between psychosis and a higher bias for empathizing over systemizing than that found in other individuals with ASD. It should be noted, however, that individuals with psychotic illness who have ASD cannot be characterised as having a bias towards empathizing when compared to general population norms: they all still show a Balanced, Type S, or Extreme Type S cognitive style. This must be considered in any future theoretical model of the relationship between ASD and psychosis.

More research is needed in clinical populations to further test Brosnan’s theories regarding the role of psychosis and/or mania/hypomania as a manifestation of the EFB [15, 46–47] before it is established that the link between these constructs in the general population can be extended to our understanding of the development of psychopathology. It should also be noted that the EFB itself may not lead to any psychiatric condition, since heightened empathizing could be a mostly positive trait, and reduced systemizing can be easily compensated for. The evidence from our study suggests that, as Brosnan et al. [15] reported, it may be that some features of psychotic illness, in particular features of mania/hypomania, are associated with differences in EB. IQ also plays an important role. However, psychotic illness is not a unitary construct and presents differently in affected individuals. It would be inaccurate to say that our evidence supports the idea that psychosis, as a broad collection of behaviors and thought patterns, is diametrically opposite to ASD, similarly a complex and multifaceted collection of behaviors. Some element of each of these conditions may be affected by a bias for empathizing over systemizing, and the causes of these conditions themselves may shape the bias for systemizing or empathizing. Exactly how these elements interact, and any argument for a causal role of EB in psychotic illness or any other mental health condition, requires further research.

## Supporting Information

S1 Dataset Full study data set. This is in comma-separated variable format. All variables are clearly labelled within the file.
(CSV)

## References

[1] Baron-Cohen S. The extreme male brain theory of autism. TRENDS Cogn Sci. 2002; 6(6): 248–54. 12039606 10.1016/s1364-6613(02)01904-6

[2] Asperger H. Die autistischen psychopathen’ im kindesalter. Arch Psychiat Nervenkr. 1944;117,:76–136.

[3] Baron-Cohen S, Richler J, Bisarya D, Gurunathan N, Wheelwright S. The systemizing quotient: an investigation of adults with Asperger syndrome or high-functioning autism, and normal sex differences. Philos T Roy Soc B. 2003; 358: 361–74. 12639333 10.1098/rstb.2002.1206 PMC1693117

[4] Baron-Cohen S, Wheelwright S. The Empathy Quotient: an investigation of adults with Asperger syndrome or High Functioning Autism, and normal sex differences. J Autism Dev Disord. 2004; 34(2): 163–75. 15162935 10.1023/b:jadd.0000022607.19833.00

[5] Baron-Cohen S, Cassidy S, Auyeung B, Allison C, Achoukhi M, Robertson S, et al Attenuation of Typical Sex Differences in 800 Adults with Autism vs. 3,900 Controls. PloS One. 2014; 9(7): e102251 10.1371/journal.pone.0102251 25029203 PMC4100876

[6] Baron-Cohen S, Lombardo MV, Auyeung B, Ashwin E, Chakrabarti B, Knickmeyer R. Why are autism spectrum conditions more prevalent in males? PLoS Biology. 2001; 9(6): e1001081 10.1371/journal.pbio.1001081 21695109 PMC3114757

[7] Bejerot S, Eriksson JM, Bonde S, Carlström K, Humble MB, Eriksson E. The extreme male brain revisited: gender coherence in adults with autism spectrum disorder. Brit J Psychiat. 2012; 201(2):116–23. 10.1192/bjp.bp.111.097899 22500012

[8] Lai MC, Lombardo MV, Suckling J, Ruigrok AN, Chakrabarti B, Ecker C, et al Biological sex affects the neurobiology of autism. Brain. 2013; 136(9): 2799–815. 23935125 10.1093/brain/awt216 PMC3754459

[9] Baron-Cohen S, Auyeung B, Nørgaard-Pedersen B, Hougaard DM, Abdallah MW, Melgaard L, et al. Elevated fetal steroidogenic activity in autism. Mol Psychiat. 2014. Advanced online publication. 10.1038/mp.2014.48 PMC4184868 24888361

[10] Dammann G. Borderline personality disorder and theory of mind: an evolutionary perspective In: Brüne M, Ribbert H, Schiefenhövel W, editors. The Social Brain: Evolution and Pathology. Chichester, UK: John Wiley & Sons; 2003 p. 373–417.

[11] Zahn-Waxler C, Shirtcliff EA, Marceau K. Disorders of childhood and adolescence: gender and psychopathology. Ann Rev Clin Psychol. 2008; 4: 275–303. 18370618 10.1146/annurev.clinpsy.3.022806.091358

[12] Crespi B, Badcock C. Psychosis and autism as diametrical disorders of the social brain. Behav Brain Sci. 2008; 31: 241–61. 10.1017/S0140525X08004214 18578904

[13] Gazzaniga MS. The social brain: Discovering the networks of the mind New York, NY: Basic Books; 1985.

[14] Baron-Cohen S. Mindblindness: An essay on autism and theory of mind Cambridge, MA: MIT Press; 1997.

[15] Brosnan M, Ashwin C, Walker I, Donaghue J. Can an "Extreme Female Brain" be characterised in terms of psychosis? Pers Indiv Differ. 2010; 49: 738–42.

[16] Montag C, Dziobek I, Richter IS, Neuhaus K, Lehmann A, Sylla R, et al Different aspects of theory of mind in paranoid schizophrenia: Evidence from a video-based assessment. Psychiat Res. 2011; 186(2–3): 203–9. 10.1016/j.psychres.2010.09.006 20947175

[17] Crespi B, Stead P, Elliot M. Comparative genomics of autism and schizophrenia. P Nat A Sci USA. 2010; 107 (suppl. 1): 1736–41. 10.1073/pnas.0906080106 19955444 PMC2868282

[18] Starling J, Dossetor D. Pervasive developmental disorders and psychosis. Cur Psychiat Rep. 2009; 11: 190–6. 10.1007/s11920-009-0030-0 19470280

[19] Skoukaskas N, Gallagher L. Psychosis, affective disorders, and anxiety in autistic spectrum disorder: prevalence and nosological considerations. Psychopathology. 2010; 43(1): 8–16. 10.1159/000255958 19893339

[20] Fanous A, Gardner C, Walsh D, Kendler KS. Relationship between positive and negative symptoms of schizophrenia and schizotypal symptoms in nonpsychotic relatives. Arch Gen Psychiat. 2001; 58 (7): 669–73. 11448374 10.1001/archpsyc.58.7.669

[21] Schürhoff F, Laguerre A, Szöke A, Méary A, Leboyer M. Schizotypal dimensions: continuity between schizophrenia and bipolar disorders. Schiz Res. 2005; 80 (2–3): 235–42. 10.1016/j.schres.2005.07.009 16169190

[22] Langdon R, Coltheart M. Mentalising, schizotypy, and schizophrenia. Cognition. 1999; 71 (1): 43–71. 10394709 10.1016/s0010-0277(99)00018-9

[23] Dinsdale NL, Hurd PL, Wakabayashi A, Elliot M, Crespi BJ. How are autism and schizotypy related? Evidence from a non-clinical population. PLoS ONE. 2013; 8 (5): e63316 10.1371/journal.pone.0063316 23691021 PMC3655150

[24] Russell-Smith SN, Bayliss DM, Maybery MT, Tomkinson RL. Are the autism and positive schizotypy spectra diametrically pposed in empathizing and systemizing? J Autism Dev Disord. 2013; 43 (3): 695–706. 10.1007/s10803-012-1614-9 22829244

[25] Davies W, Isles AR. Genomic imprinting and disorders of the social brain; shades of grey rather than black and white. Behav Brain Sci. 2008; 31(03): 265–6.

[26] Lord C, Rutter M, Goode S, Heemsbergen J, Jordan H, Mawhood L, et al Austism diagnostic observation schedule: A standardized observation of communicative and social behavior. J Autism Dev Disord. 1989; 19(2): 185–212. 2745388 10.1007/BF02211841

[27] Lord C, Rutter M, Le Couteur A. Autism Diagnostic Interview-Revised: A revised version of a diagnostic interview for caregivers of individuals with possible pervasive developmental disorders. J Autism Dev Disord. 1994; 24(5): 659–85. 7814313 10.1007/BF02172145

[28] Castle DJ, Jablensky A, McGrath JJ, Carr V, Morgan V, Waterreus A, et al The diagnostic interview for psychoses (DIP): development, reliability and applications. Psychol Med. 2006; 36: 69–80. 16194284 10.1017/S0033291705005969

[29] McGuffin P, Farmer A, Harvey I. A polydiagnostic application of operational criteria in studies of psychotic illness: development and reliability of the OPCRIT system. Arch Gen Psychiat. 1991; 48(8): 764 1883262 10.1001/archpsyc.1991.01810320088015

[30] Prosser H, Moss S, Costello H, Simpson N, Patel P, Rowe S. Reliability and validity of the Mini PAS-ADD for assessing psychiatric disorders in adults with intellectual disabilities. J Intell Disabil Res. 1998; 42(4): 264–72. 10.1046/j.1365-2788.1998.00146.x 9786440

[31] American Psychiatric Association. Diagnostic and statistical manual of mental disorders (4th edition, text revision ed.) Washington, DC: American Psychiatric Association; 2000.

[32] World Health Organisation. International Statistical Classification of Diseases and Related Health Problems. 2007. Retrieved 08/09/2014, from http://apps.who.int/classifications/apps/icd/icd10online/

[33] Spitzer RL, Endicott J, Williams JB. Research diagnostic criteria. Arch Gen Psychiat. 1979; 36(12): 1381–3. 496556 10.1001/archpsyc.1979.01780120111013

[34] Larson FV, Wagner AP, Jones PB, Tantam D, Lai M-C, Baron-Cohen S, et al. Psychosis in autism: phenomenology and clinical features. 2015; manuscript in submission.

[35] Wakabayashi A, Baron-Cohen S, Wheelwright S, Goldenfeld N, Delaney J, Fine D, et al Development of short forms of the Empathy Quotient (EQ-Short) and the Systemizing Quotient (SQ-Short). Pers Indiv Differ. 2006; 41: 929–40.

[36] Wheelwright S, Baron-Cohen S, Goldenfeld N, Delaney J, Fine D, Smith R, et al Predicting autism spectrum quotient (AQ) from the systemizing quotient-revised (SQ-R) and empathy quotient (EQ). Brain Res. 2006; 1079(1): 47–56. 16473340 10.1016/j.brainres.2006.01.012

[37] Baron-Cohen S, Wheelwright S, Robinson J, Woodbury-Smith M. The Adult asperger assessment (AAA): a diagnostic method. J Autism Dev Disord. 2005; 35(6): 807–19. 16331530 10.1007/s10803-005-0026-5

[38] Lai MC, Lombardo MV, Ruigrok ANV, Chakrabarti B, Wheelwright SJ, Auyeung B, et al Cognition in Males and Females with Autism: Similarities and Differences. PLoS ONE. 2012; 7(10): e47198 10.1371/journal.pone.0047198 23094036 PMC3474800

[39] Lai MC, Lombardo MV, Chakrabarti B, Ecker C, Sadek SA, Wheelwright SJ, et al Individual differences in brain structure underpin empathizing–systemizing cognitive styles in male adults. Neuroimage. 2012; 61(4): 1347–54. 10.1016/j.neuroimage.2012.03.018 22446488 PMC3381228

[40] Lai MC, Lombardo MV, Pasco G, Ruigrok AN, Wheelwright SJ, Sadek SA, et al A behavioral comparison of male and female adults with high functioning autism spectrum conditions. PLoS ONE. 2011; 6(6): e20835 10.1371/journal.pone.0020835 21695147 PMC3113855

[41] Wechsler D. Wechsler Abbreviated Scale of Intelligence San Antonio, TX: The Psychological Corporation; 1999.

[42] O'Connor BP. All-in-one programs for exploring interactions in moderated multiple regression. Educ Psychol Meas. 1998; 58: 833–7.

[43] Aiken LS, West SG. Multiple regression: testing and interpreting interactions Newbury Park, CA: Sage; 1991.

[44] Cohen J, Cohen P. Applied multiple regression/correlation analysis for the behavioral sciences (2nd Ed.). Hillsdale, NJ: Erlbaum; 1983.

[45] Goldenfeld N, Baron-Cohen S, Wheelwright S. Empathizing and systemizing in males, females, and autism. Clin Neuropsychiat. 2005; 2(6): 338–45.

[46] Brosnan M, Chapman E, Ashwin C. Adolescents with Autism Spectrum Disorder Show a Circumspect Reasoning Bias Rather than ‘Jumping-to-Conclusions’. J Autism Dev Disord. 2013; 44(3): 513–20. 10.1007/s10803-013-1897-5 24002414

[47] Brosnan M, Ashwin C, Gamble T. Greater Empathizing and reduced Systemizing in people who show a jumping to conclusions bias in the general population: Implications for psychosis. Psychosis. 2013; 5(1): 71–81.

[48] Hallerback MU, Lugnegard T, Gillberg C. Is autism spectrum disorder common in schizophrenia? Psychiat Res. 2012; 198(1): 12–7. 10.1016/j.psychres.2012.01.016 22421071

[49] Baron-Cohen S, Wheelwright S, Skinner R, Martin J, Clubley E. The Autism-Spectrum Quotient (AQ): Evidence from Asperger syndrome/high-functioning autism, males and females, scientists and mathematicians. J Autism Dev Disord. 2001; 31(1): 5–17. 11439754 10.1023/a:1005653411471

[50] Konstantareas MM, Stewart K. Affect regulation and temperament in children with autism spectrum disorder. J Autism Dev Disord. 2006; 36 (2): 143–54. 16456727 10.1007/s10803-005-0051-4

[51] Samson AC, Huber O, Gross JJ. Emotion regulation in Asperger’s syndrome and high-functioning autism. Emotion. 2012; 12 (4): 659–65. 10.1037/a0027975 22642342

[52] Tantam D. Asperger syndrome in adulthood In: Frith U, editor. Autism and Asperger Syndrome. Cambridge, UK: Cambridge University Press, 1991 p. 147–183.

[53] Lai MC, Lombardo MV, Chakrabarti B, Baron-Cohen S. Subgrouping the autism “spectrum”: reflections on DSM-5. PLoS Biology. 2013; 11(4): e1001544 10.1371/journal.pbio.1001544 23630456 PMC3635864

[54] Ousley O, Cermak T. Autism Spectrum Disorder: Defining Dimensions and Subgroups. Curr Dev Disord Rep. 2014; 1(1): 20–8. 10.1007/s40474-013-0003-1 25072016 PMC4111262

